# The biological function of integrin-linked kinase on bone formation

**DOI:** 10.1016/j.bonr.2025.101834

**Published:** 2025-03-10

**Authors:** Yu-ling Liu, Yue-ming Mei, Jing-qiong Xun, Zhuo-yue Lv, Qian He, Zhou-bo-ran Liu, Lin Li, Fen Xie, Ru-chun Dai

**Affiliations:** a National Clinical Research Center for Metabolic Diseases, Hunan Provincial Key Laboratory for Metabolic Bone Diseases, Department of Metabolism and Endocrinology, The Second Xiangya Hospital of Central South University, Changsha 410011, Hunan, China; b Medicine School, Changsha Social Work College, Changsha 410004, Hunan, China; c Department of Endocrinology and Metabolism, The Affiliated Changsha Hospital of Xiangya School of Medicine, Central South University, Changsha 410005, Hunan, China

**Keywords:** Integrin-linked kinase (ILK), Osteoblastogenesis, BMSCs, Osteoblasts, OP, Angiogenesis, Bone remodeling

## Abstract

Bone remodeling process is the basis for maintaining normal bone microstructure and promoting fracture repair. Recent studies have proven that integrins can promote bone formation and fracture repair. Integrin-linked kinase (ILK), as the proximal effector of the integrin receptor, is a key protein factor linking integrin and cytoskeleton. It is involved in crucial cellular processes including proliferation, survival, differentiation, migration, invasion, and angiogenesis reflects on systemic changes in the kidney, heart, muscle, skin, and vascular system. At present, the regulation effect of ILK in bone formation attracts the attention of researchers. This review emphasizes that ILK as a key molecule affects the functions of bone marrow stromal cells (BMSCs) and osteoblasts, and regulates bone formation. Additionally, ILK plays a key role in the process of“angiogenic–osteogenic coupling ”. The present role of ILK in the pathogenesis of osteoporosis is also described. Strategies that target ILK may as a new prospective treatment for osteoporosis (OP).

## Highlights

• ILK serves as a key molecule that affects the function of BMSCs and osteoblasts via MAPK, BMP, and Wnt signaling pathways.
• ILK is involved in osteoporosis pathogenesis, and ILK-targeted therapies may emerge as a novel treatment for OP.
• ILK may play a crucial role in the process of “angiogenic–osteogenic coupling”.

## 1 Introduction

Bone is a special structural organ that requires the balance of bone formation and bone resorption. From birth to death, bones undergo a continuous process of modeling and remodeling. The bone remodeling cycle encompasses seven distinct stages: quiescence, activation, reabsorption, reversal, formation, mineralization, and termination. During this process, osteoblasts and osteoclasts play a pivotal role. Osteoclasts are responsible for bone resorption, which involves the removal of mineralized bone. Subsequently, osteoblasts form the bone matrix after undergoing mineralization. The processes of bone resorption and bone formation are tightly coupled, and bone formation accompanies each cycle of bone resorption to maintain bone integrity. If the balance of this process is broken, bone disease will occur. Osteoporosis (OP) is a metabolic bone disease characterized by reduced bone mass, destroyed microarchitecture of bone, increased bone brittleness, and easy fracture (Kalb et al., 2013). OP tends to occur in postmenopausal women mainly because of estrogen deficiency, and in men, the decrease in bone formation with increasing age is the primary cause (Consensus development conference, 1993; Compston et al., 2019).

During bone formation process, the integrin-dependent interaction with the developing bone matrix and the participation of downstream signaling factors are all necessary for bone growth (McDonald et al., 2008). Integrin mediates the direct interaction between osteoblasts and extracellular matrix (ECM) and plays a crucial role in regulating osteoblast differentiation (Moursi et al., 1997). beta1 integrin is involved in osteoblast mineralization (Brunner et al., 2011). Moreover, integrins may initiate intracellular signal transduction to activate the cellular response of bone cells to dynamic fluid flow, and may act as a bone mechanical sensor (Litzenberger et al., 2010). In addition, Phillips JA et al. provided evidence that integrins in osteocytes mediate mechanical transduction (Phillips et al., 2008). Inhibition of integrin disrupts osteocyte cellular processes, as well as their mechanosensation and mechanotransduction (Haugh et al., 2015). These studies indicate that integrins are essential for osteoblast differentiation and mechanosensation and mechanotransduction of osteocytes. Integrin αv/β1 acts as a receptor for osteopontin (OPN) to affect the bone marrow stromal cells (BMSCs) (Chen et al., 2014). The activation of integrin alpha5 is sufficient to enhance ERK1/2-MAPK and PI3K signaling, thereby promoting osteogenic differentiation of human BMSCs (Hamidouche et al., 2009). Targeted drugs targeting the key integrins expressed by BMSCs can improve bone microstructure and increase bone mass by promoting osteoblast differentiation (Marie, 2013). These studies demonstrate that integrins play a crucial role in regulating mechanical conduction, osteogenic differentiation, and bone formation within the osteoblast lineage. Moreover, integrins have long been considered to mediate bone resorption by regulating osteoclast attachment, cytoskeletal organization, and polarization (Nakamura et al., 2007; Feng and Teitelbaum, 2013).

Integrin-linked kinase (ILK), a key protein factor linking integrin and cytoskeleton, has been shown to play important regulatory roles in bone formation and osteoblastogenesis (Dejaeger et al., 2017). The serine/threonine protein kinase, identified by Hannigan in 1996 through a yeast two-hybrid screening system utilizing the cytoplasmic region of the integrin β1 subunit as bait, is thought to play a role in cell adhesion and anchorage-dependent cell growth (Hannigan et al., 1996). The discovery of ILK is an important milestone in the field of biomedical research, providing new perspectives and tools for understanding how the extracellular matrix influences cellular behavior through integrins. It is a 59 kDa protein containing a phosphoinositide phospholipid-binding domain flanked by an N-terminal ankyrin repeat domain and a C-terminal serine/threonine protein kinase domain (Persad and Dedhar, 2003). It has been primarily detected at focal adhesion sites (Li et al., 1999), confirmed by co-localization with focal adhesion markers like vinculin and paxillin (Nikolopoulos and Turner, 2001). ILK is a highly evolutionarily conserved intracellular protein that was originally identified as an integrin-interacting protein, and its expression is vital during both embryonic development and tissue homeostasis (Hannigan et al., 2011). After that, some researchers also discovered that ILK combined with PINCH played an important role in cell adhesion, growth factor, and Wnt signaling pathways (Tu et al., 1999). ILK binds to a cysteine and histidine-rich protein (PINCH) and parvin to form a ternary complex, called the IPP complex (ILK-PINch-parvin), which regulates cell adhesion and cytoskeleton formation (Ghatak et al., 2013; Tu et al., 2001). ILK also interacts with many proteins and regulates multiple signaling pathways, such as PI3K/AKT, glycogen nuclear factor-kappa B (NF-κB), synthase kinase 3-beta (GSK3β), mammalian target of rapamycin (mTOR), cell division control protein 42 homolog (Rac/Cdc42), Snail1/E-cadherin and vascular endothelial growth factor (VEGF) (Liang et al., 2014; Shen et al., 2016; Yan et al., 2014; Zhao et al., 2013; Wani et al., 2011; Tan et al., 2004). These pathways regulate cell apoptosis, proliferation and mitosis, migration, invasion, cell adhesion-mediated cell survival (anchor-dependent cell death), and angiogenesis and tumor angiogenesis.

Over the years, ILK has been reported to be involved in a variety of pathophysiological processes. ILK dysregulation is associated with a variety of human cancers (McDonald et al., 2008). In contemporary research, upregulation of ILK expression occurs in many human malignant tumors and has a strong correlation with poor prognosis, thus ILK has been more studied in tumor diagnosis and prognosis. It was found that ILK overexpression is significantly associated with distant metastasis of osteosarcoma (Rhee et al., 2013). Under physiological conditions, ILK plays a crucial role in the cardiovascular system, particularly in the formation of new blood vessels and cardiomyogenesis (Traister et al., 2012; Hannigan et al., 2007; Traister et al., 2014). ILK has no apparent tissue or cell line specificity. However, in muscle cells, especially smooth muscle cells, the expression level of ILK was significantly elevated (Yamaji et al., 2001). Analyzing the latest data on ILK expression in human tissues, the consensus normalized expression (NX) levels of ILK in bone marrow is 7.5, and protein expression levels were assessed as medium (Gorska and Mazur, 2022). Our group also confirmed this result.

The number of therapeutic molecules that stimulate bone formation is currently limited. In this regard, the present study underscores the pivotal role of integrins in osteoblast differentiation and highlights the potential therapeutic benefits of targeting integrins within osteoblast lineage cells to enhance bone formation in vivo. This review summarizes that ILK can modulate the process of bone formation and is implicated in the development of osteoporosis. Moreover, drugs targeting ILK have garnered interest as potential bone anabolic therapies. Despite the attention that ILK has received over the past nearly three decades, a variety of conflicting findings and opinions have led to the fact that some of the key aspects of ILK regulation of bone formation remain controversial or unestablished. For deeper understanding, our review reveals the regulation and role of ILK in bone formation and provides an overview of potential therapeutic approaches targeting ILK in various bone diseases.

## 2 Role of ILK in BMSCs

Osteogenic differentiation of BMSCs plays an essential role in bone formation, BMSCs are the common precursors of osteoblasts and adipocytes. Changes in the two differentiation pathways affect the level of bone remodeling. Its imbalance can lead to OP. Many factors work together to construct the osteogenic microenvironment, regulate the migration, proliferation, and differentiation of BMSCs, and finally complete the process of bone formation (Alliston and Derynck, 2002). ILK has been identified as the proximal effector of the integrin receptor (Dedhar et al., 1999). Integrin-mediated signals regulate gene expression, differentiation, and survival of osteoblasts and BMSCs, and play a key role in bone formation and repair (Marie, 2013; Bouvard et al., 2013). ILK's role in BMSCs has gradually become clear. Global transcriptome analysis found that the extracellular matrix proteins in BMSCs increased significantly, and the upregulated proteins are most likely to be integrin signaling and ILK signaling, compared with in hematopoietic stem cells (HSC), human embryonic stem cells (hESC), and fibroblasts (Ren et al., 2011). The author also believes that BMSCs use integrin and its signaling to move and proliferate. Subsequently, a study confirmed the important role of ILK in BMSCs proliferation by stimulating BMSC proliferation when ILK was overexpressed in vitro (Huang et al., 2017). Moreover, ILK inactivation via siRNA has been shown to diminish the osteogenic differentiation of BMSCs, and the impaired osteogenic differentiation phenotype was accompanied by a significant inhibition of p38 phosphorylation by more than 50 % (Yue et al., 2019). In a recent study in 2024, Z. Huang et al. showed that the osteogenic differentiation capacity of human BMSCs was significantly reduced after ILK antagonist treatment, and the mechanism was the regulation of Nuclear β-catenin expression through the Akt/GSK-3β pathway (Huang et al., 2024). It has also been proved that periostin enhances the osteogenic differentiation of BMSCs in ovariectomized(OVX) rats via the ILK/pAkt/pGSK3β axis (Liu et al., 2021). The osteogenic differentiation ability of Pinch-deficient BMSCs decreases and tends to adipogenic differentiation, the mechanism may be through down-regulation of ILK expression (Wang et al., 2019). These observations implicate ILK in the control of proliferation and osteogenic differentiation of BMSCs. Currently, there are few studies on the deeper mechanisms of ILK and BMSCs. Thus, our team is currently conducting a study on the correlation between ILK and BMSCs, with samples derived from human subjects. Preliminary, unpublished data indicate that ILK significantly enhances the osteogenic differentiation of BMSCs. We aim to delve deeper into the underlying molecular mechanisms driving this process.

## 3 Role of ILK in osteoblasts

Osteoblasts, the cells that build our bones, are very versatile and important cells that need to be tightly regulated at all stages of their differentiation to ensure normal bone development and homeostasis. The first study to describe the impact of ILK on osteoblast function was published in 2014 (El-Hoss et al., 2014). Specifically, ILK knockout in mouse osteoblasts resulted in increased mineralization, accompanied by upregulated expression of Runx2, Collagen I, and Bone Sialoprotein (Bsp). Dejaeger M et al. also reported in 2017 that the bone mass of trabecular bone in ILK-conditional knockout mice significantly decreased after five weeks of age and persisted into adulthood in male mice (Dejaeger et al., 2017). They conducted in vitro experiments to confirm that the absence of ILK can impair collagen matrix production and mineralization. The core mechanism involves the loss of ILK in primary osteoblasts, which results in poor binding of cell-matrix interactions to the cytoskeleton. This, in turn, damages the organization, adhesion, diffusion, and migration of F-actin. Moreover, ILK deficiency in osteoblasts impairs the classical BMP and Wnt/β-catenin signaling pathway. The underlying mechanism was that ILK knockdown reduced the phosphorylation of Smad1/5/8 in response to BMP2 stimulation. Meanwhile, ILK knockout resulted in a non-responsive state to the regulation of GSK-3β, resulting in a decrease in the accumulation of b-catenin and the Wnt target gene expression cannot to be activated. In terms of ILK as a mechanism of osteogenic differentiation, the ILK pathway can mediate targeted differentiation signals from topographical cues to osteoblasts and provide new targets for the modification and biofunctionalization of biomaterials (Wang et al., 2013). The main mechanism is that ILK activates the β-catenin signaling pathway by increasing β-catenin expression and inhibits GSK3β degradation by phosphorylation. In 2015, a study showed that ILK can be activated by insulin to regulate alkaline phosphatase and collagen synthesis in osteoblast cells (Yoon et al., 2015). In 2019, Y. Wang et al. found that the inactivation of Pinch in mature osteoblasts causes bone loss, the potential mechanism for this is a reduction in the expression of ILK (Wang et al., 2019). ILK itself acts as part of a key pathway for the mechanism of this studied phenotype. Therefore, ILK plays significant and context-specific roles in regulating osteoblast function.

## 4 ILK signaling in osteoblastogenesis

The relationship between ILK and several conserved signaling pathways involved in the regulation of osteoblastogenesis suggests a role for ILK in bone formation. ILK has been shown to affect several key intracellular signal transduction pathways either indirectly or directly, either directly as a kinase or indirectly as a protein through protein interactions.

Mitogen-activated protein kinases (MAPKs) pathway is crucial for the regulation of various cellular metabolism processes, such as cell differentiation, morphology, and mobility. MAPKs consist of three signaling pathways: p38, extracellular signal-regulated kinase 1/2 (ERK1/2), and c-Jun amino-terminal kinase (JNK). In prior research, it has been demonstrated that ILK activates the p38 MAPK pathway via phosphorylation (Smeeton et al., 2010). Nevertheless, the activation of the p38 MAPK pathway does not rely on the kinase activity of ILK. G. Yue et al. identified the activation of p38 MAPK signaling pathway by ILK as a regulator of osteogenic differentiation in BMSCs (Yue et al., 2019). In a study published in Acta Biomaterialia in 2014, the roles of P38, ERK1/2, and JNK were confirmed, revealing that ILK activates both P38 and ERK1/2 to enhance osteoblast differentiation while having no impact on the JNK pathway (Wang et al., 2014).

Moreover, ILK can modulate BMP and Wnt signaling in osteoprogenitor cells, both known as central mediators of osteoblast differentiation and bone formation (Dejaeger et al., 2017; Salazar et al., 2016; Baron and Kneissel, 2013). As we all know, BMPs are crucial regulators of osteogenesis. ILK has been shown to affect the expression of BMP-2 and BMP-4 to regulate osteoblast differentiation and mineralization (Su et al., 2010; Tan et al., 2012; Tseng et al., 2010). The signaling cascade regulated by BMP-2 can activate osteoblast differentiation and bone matrix mineralization (Salazar et al., 2016). Moreover, the BMP pathway can interact with integrins to form cross-talk (Ashe, 2016). In another study, ILK mediated Wnt/β-catenin signaling can activate BMSCs osteogenic differentiation (Lu et al., 2020). The ILK/β-catenin signaling pathway is also involved in osteogenic differentiation (Wang et al., 2013).

**Fig. 1:**
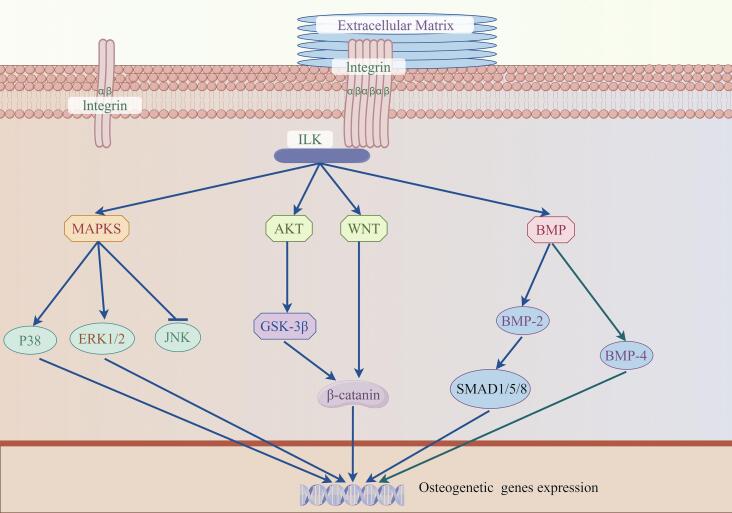
ILK-mediated signals that regulate osteoblastogenesis. Three major signaling mechanisms are induced by ECM–integrin-ILK interactions, including MAPK, BMP, and Wnt signaling. (Created with Figdraw).

Besides, recent investigations have elucidated the regulatory role of ILK in osteogenic differentiation, affecting both human and rat-derived BMSCs, via modulation of the Akt/GSK-3β pathway (Huang et al., 2024; Liu et al., 2021). In MC3T3-E1 cells, studies have also confirmed that the knockdown of ILK impairs the phosphorylation of GSK-3β at Ser9 (El-Hoss et al., 2014). Furthermore, GSK-3β has been demonstrated for migration and cytoskeletal dynamics in a variety of cells (Sun et al., 2009). Such insights propose that ILK could be a key element in triggering osteoblastogenesis. Owing to its cellular positioning, ILK is adept at interfacing extracellular alterations with a multitude of intracellular processes. This includes MAPK, BMP, and Wnt signaling circuits, which are also associated with osteoblastogenesis pathways (Fig. 1). Consequently, through its interaction with diverse signaling pathways, ILK assumes a significant function in bone formation.

## 5 ILK signaling in vascularization

In recent years, vascularization has attracted more and more attention in the pathogenesis of osteoporosis. The pathogenesis of osteoporosis consists of three main factors, including excessive bone resorption, insufficient bone formation, and insufficient vascularization (Cui et al., 2022). There is no doubt that angiogenesis plays an important role in the remodeling of osteogenesis (Watson and Adams, 2018). Blood vessels not only deliver nutrients, oxygen, growth factors, and hormones to reach bone tissue but also regulate bone formation (Hankenson et al., 2011; Zheng et al., 2018). “Angiogenesis-osteogenesis coupling” refers to the close spatiotemporal link between osteogenesis and angiogenesis (Maes, 2013; Lafage-Proust et al., 2010; Grosso et al., 2017; Zitron and Hawley, 1989; Schoenly et al., 1991). In particular, a newly discovered vascular subtype (H-type vessels) regulates the growth of bone blood vessels, recruits bone progenitor cells, and combines osteogenesis with angiogenesis (Kusumbe et al., 2014).

ILK not only stimulates the secretion of angiogenic factors but also promotes the proliferation and migration of endothelial cells and inhibits their apoptosis, thus regulating angiogenesis (Zhou and Li, 2017). Hypoxia-inducible factor 1 alpha (HIF-1α) and Vascular endothelial growth factor (VEGF) are important regulatory factors that initiate H-type vessels angiogenesis. During angiogenesis, the expression of VEGF and HIF-1α is regulated by ILK (Tan et al., 2004; Moody et al., 1991). Silencing of ILK markedly inhibits the proliferation, migration, and angiogenic capacity of primary human scar microvascular endothelial cells (HSMECs), thus, ILK regulates HSMEC proliferation and angiogenesis (Li et al., 2016a).In 2019, research published in Nature Communications showed that ILK can directly control retinal angiogenesis through the Wnt signaling pathway (Park et al., 2019). ILK gene therapy improves cardiac remodeling and function after myocardial infarction in rats and pigs by increasing angiogenesis (Ding et al., 2009; Mao et al., 2014). Moreover, ILK can partially regulate the expression of VEGF in mesenchymal stem cells (MSCs) through the AKT and mTOR signaling pathway, which indirectly plays a role in promoting angiogenesis (Zeng et al., 2017). There is a lack of research indicating an association between ILK and H-vessels. Considering the established connection between ILK, bone formation, and angiogenesis, it is prudent to investigate the potential role of ILK in the “Angiogenesis-osteogenesis coupling” process. Since the current clinical drugs for osteoporosis are limited to promoting bone formation and resorption, it is necessary to clarify the specific function and molecular mechanism of ILK in type H vessels. Combined with previous studies on the role of ILK in bone formation, ILK may serve as a novel target for a drug that promotes both bone formation and type H angiogenesis, thus contributing to the transformation of a promising dual-target drug that promotes bone formation.

Drawing upon existing literature, we speculated that ILK can promote osteogenic differentiation (Table 1), and may regulate the coupling between angiogenesis and bone formation by regulating the expression of VEGF in BMSCs, possibly through the Wnt/β-catenin pathway. In the context of bone resorption, ILK-deficient osteoclasts may exhibit functional impairments due to various downstream mechanisms without affecting their differentiation process (Dossa et al., 2010). Conversely, as previously noted, certain studies have observed increased mineralization when ILK is inactivated in osteoblasts. This discrepancy underscores the need for further investigation to conclusively determine ILK's regulatory influence on bone formation.

**Table 1:** Summary of the relevant studies showing the role of ILK in Osteogenic differentiation.

Species	Cell types	Vitro or vivo	Methods	Pathways	Observed functions of ILK	Year	References
Human	BMSCs	Vitro	inhibition	Akt/GSK-3β/β-catenin	Osteogenic differentiation	2024	(Huang et al., 2024)
Rats	BMSCs	Vitro	Overexpression	Akt/GSK-3β	Osteogenic differentiation	2021	(Liu et al., 2021)
Rats	BMSCs	Vitro	Knockdown	Wnt/β-catenin	Osteogenic differentiation	2020	(Lu et al., 2020)
Rats	BMSCs	Vitro	Knockdown	p38	Osteogenic differentiation	2019	(Yue et al., 2019)
Mice	BMSCs	Vivo	Knockout	Unknown	Bone homeostasis	2019	(Wang et al., 2019)
Rats	BMSCs	Vitro	Overexpressed	Unknown	Proliferation	2017	(Huang et al., 2017)
Mice	osteoprogenitors	Vivo	Knockout	BMP, Wnt/b-catenin	osteoblast function, cell-matrix interactions, Bone homeostasis	2017	(Dejaeger et al., 2017)
Rats	UMR-106 cells	Vitro	Stimulated by Insulin	Akt	Collagen synthesis, Alkaline phosphatase activity	2015	(Yoon et al., 2015)
Mice	MC3T3-E1 cells	Vitro and vivo	Knockdown and Knockout	GSK3β	Mineralization	2014	(El-Hoss et al., 2014)
Human	MG63 cells	Vitro	Knockdown	ERK1/2, p38	Osteoblast differentiation	2014	(Wang et al., 2014)
Human	MG63 cells	Vitro	Knockdown	β-catenin	Differentiation	2013	(Wang et al., 2013)
Mice	osteoblasts	Vitro	Knockdown	BMP-4	Bone nodule formation, Bone mineralization	2012	(Tan et al., 2012)
Mice	Osteoclasts	Vitro and vivo	Knockout	Unknown	Osteoclast function	2010	(Dossa et al., 2010)
Mice	MC3T3-E1, osteoblasts	Vitro	Knockdown	BMP-2	Proliferation, Osteoblastic differentiation	2010	(Su et al., 2010)
Human, Mice	MG-63, hFOB, M2-10B4	Vitro	Knockout	BMP-2	Unknown	2010	(Tseng et al., 2010)

## 6 Role of ILK in osteoporosis

While numerous studies have explored the relationship between ILK and bone metabolism, the precise role of ILK in osteoporosis remains elusive. In 2010, Meury T et al. conducted a study to prove that ILK is related to osteoporosis (Meury et al., 2010). Their research indicated that ILK may be related to osteoporosis because mutations in the ILK phosphate receptor site in the alpha (alpha NAC) transcriptional regulator can affect matrix gene expression and lead to osteopenia (Meury et al., 2010). The mutation leads to a reduction in the number of nuclear alpha NAC and reduced occupancy of the osteocalcin gene promoter, resulting in a significant decrease in osteocalcin gene transcription, and secondly a decrease in type I collagen expression. Additionally, the mutation accelerates mineralization and promotes immature woven bone formation, ultimately resulting in a reduced bone mass phenotype. Dossa T et al. discovered an increase in bone mass and trabecular thickness following the specific knockdown of ILK in mouse osteoclasts (Dossa et al., 2010). Another study demonstrated that the loss of ILK in osteoprogenitor cells in vivo results in impaired postnatal bone acquisition in mice, leading to reduced bone mass in males and decreased osteogenic growth in females (Dejaeger et al., 2017). Mechanistically, this study elucidates that impaired BMP and Wnt/β-catenin signaling in the ILK cKO model leads to a loss of function of metabolic signaling through these pathways for bone formation, which ultimately leads to the acquisition and maintenance of insufficient bone mass for the development of osteoporosis. Additionally, it has been demonstrated that ILK signaling status may be an important modifier of ER signaling (Acconcia et al., 2006). Therefore, it is worthwhile to explore whether there is a correlation between ILK and osteoporosis in postmenopausal women due to the decline of estrogen. Our research has revealed that the downregulation of ILK expression within the bone marrow microenvironment of osteoporosis patients offers novel insights into potential therapeutic targets for this condition (Zhou et al., 2019). Xie Y et al. also found that ILK expression profiles of circulating exosomes isolated from patients with clinically different degrees of bone loss could be used as prognostic markers for predicting changes in bone quality (Xie et al., 2018). These studies demonstrate the promising value of ILK as a marker to supplement the diagnosis and prognostic assessment of primary osteoporosis.

The bone marrow microenvironment is formed by the communication of soluble factors produced by different cells in the bone marrow (Li et al., 2016b). It is well known that BMSCs are located in the bone marrow microenvironment and are the common precursor of osteoblasts and adipocytes. The imbalance of their differentiation into the two lineages is of great significance in the etiology of bone diseases such as osteoporosis. In the previous article, we have summarized that ILK can promote the proliferation and osteogenic differentiation of BMSCs. However, only a few studies have cautiously explored the role of ILK during adipogenesis in stem cells (Goessler et al., 2008; Tseng and Kolonin, 2016; Loibl et al., 2016). It has been demonstrated that ILK expression is upregulated in stem cells from both bone marrow and adipose tissue during chondrogenic differentiation (Goessler et al., 2008). Moreover, some studies have found a relationship between ILK and adipocytes (de Frutos et al., 2023; de Frutos et al., 2022; Wang et al., 2011). These publications highlighted that ILK overexpression inhibits adipogenesis. Increased ILK expression and activity in adipocytes reduces adipogenesis and promotes adipocyte transdifferentiation (de Frutos et al., 2022). Therefore, the decreased expression of ILK in the bone microenvironment of osteoporosis patients may lead to decreased osteogenic differentiation of BMSCs and promote adipogenesis, potentially contributing to bone mass loss.

Glucocorticoid-induced osteoporosis (GIO) is the predominant cause of secondary osteoporosis due to its inhibition of osteogenic differentiation leading to bone loss (Buckley and Humphrey, 2018; Rizzoli and Biver, 2015). Naves MA et al. reported that Glucocorticoids may cause osteoblast apoptosis, the reason may be that it inhibits the activation of ILK (Naves et al., 2011). This suggests the involvement of ILK in the pathogenesis of GIO. Additionally, the well-known pathophysiological processes that cause osteoporosis (such as ROS) can also modulate ILK, triggering downstream signaling to cause osteoporosis (Schroder, 2019; Troyano-Suarez et al., 2015). Thus, ILK may play a role in the development of secondary osteoporosis.

Exercise has a very good effect on the prevention and treatment of osteoporosis (Yuan et al., 2016). Different exercise modes will affect the expression of ILK (Sontam et al., 2015). Concurrently, multiple studies have investigated the relationship between ILK and mechanical stimulation (Maier et al., 2008; Wang et al., 1985; Andresen et al., 2022). It has been observed that mechanical stretch in vitro induces increased osteogenic differentiation and up-regulation of ILK expression in rat BMSCs (Hu et al., 2019). Therefore, the role of ILK in osteoporosis therapy deserves our attention as well. From these studies, ILK is not only involved in the pathogenesis of primary osteoporosis and secondary osteoporosis, but also in the regulation of osteoporosis by mechanical stimulation, so there is a significant space for ILK to be discovered in the pathophysiology of osteoporosis.

## 7 ILK-based strategies for bone building

Studies highlighting the pivotal role of ILK in diverse cellular functions suggest its potential as a therapeutic target for various diseases. Inhibitors of ILK could emerge as targeted therapy drugs for cancer (Zheng et al., 2019). In recent years, new methods for targeting integrins and promoting osteogenic differentiation and bone regeneration of MSCs have been developed. ILK is the core element in the regulation of integrin signaling (Yoganathan et al., 2000). Therefore, ILK has great therapeutic potential for bone diseases. At present, there are few molecular targeted drugs for osteoporosis, including RANKL inhibitors and sclerostin inhibitors. Moreover, bisphosphonates and SERMs have more adverse effects, while parathyroid hormone and related peptide analogs that stimulate bone resorption are limited to 2 years of use (Kraenzlin and Meier, 2011). If ILK is formulated as a new targeted therapy for osteoporosis, it will constitute a significant advancement in sustaining bone health. The ILK/p38 pathway can augment the osteogenic differentiation of BMSCs on amorphous carbon coating (Yue et al., 2019). Amorphous carbon (a-C) is a potential candidate for creating both osteoinductive and biocompatible surfaces on titanium implants (Yue et al., 2019). The use of a-C to coat implanted bioengineered scaffold materials has garnered attention due to its potential to enhance both BMSCs attachment and osteogenic differentiation. Therefore, the combination of ILK agonists with amorphous carbon coating on titanium implants may serve as a promising strategy to enhance bone regeneration in patients suffering from non-healing fractures. Both animal and clinical studies have demonstrated that ultrasound (US) stimulation can expedite fracture healing. It has been established that the US can stimulate nitric-oxide synthase expression, thereby promoting bone formation through the ILK pathway in osteoblasts (Tang et al., 2007). Consequently, this approach could offer an effective strategy for promoting bone formation. Our research team is also actively pursuing ILK-based research strategies to promote bone regeneration, and the preliminary results are highly promising.

## 8 Conclusions and perspectives

ILK is a complex multifunctional protein that dynamically regulates signaling from integrin-matrix interactions or growth-factor-receptor stimulation by binding intermolecular interactions and kinase activity. Taken together, the in vivo and in vitro studies described in this review suggest that ILK plays an essential role in the process of bone formation. Down-regulation of ILK in BMSCs attenuates their proliferative activity and osteogenic differentiation capacity. ILK cKO model fails to acquire and maintain adequate bone mass. Macroscopically, downregulation or inactivation of ILK expression is involved in the pathogenesis of primary and secondary osteoporosis. However, more precisely defined mechanisms need to be further explored.

In recent years, exosomal integrin has garnered significant interest, and the potential impact of exosomal ILK on bone formation warrants further investigation. Currently, ILK has not been implemented in the clinical management of bone metabolic diseases. In forthcoming years, ILK may serve as a prognostic factor for predicting changes in bone quality. Therapeutic strategies targeting ILK may improve the treatment of osteoporosis and other bone diseases by promoting a two-way linkage between bone formation and H-type angiogenesis.

## Abbreviations

ILK: Integrin-linked kinase
BMSCs: Bone marrow stromal cells
OP: Osteoporosis
ECM: Extracellular matrix
OPN: Osteopontin
ERK1/2: Extracellular signal-regulated kinase 1/2
MAPKs: Mitogen-activated protein kinases;
PI3K: Phosphatidylinositide 3-kinases
IPP complex: ILK, PINCH and parvin form complexes
AKT: Protein kinase B
NF-κB: Glycogen nuclear factor-kappa B
GSK3β: Synthase kinase 3-beta;
mTOR: Mammalian target of rapamycin
Rac/Cdc42: Cell division control protein 42 homolog
VEGF: Vascular endothelial growth factor
HSC: Hematopoietic stem cells
hESC: Human embryonic stem cells
OVX: Ovariectomy
Runx2: Runt related transcription factor2
Collagen I: Collagen type I
BMPs: Bone morphogenetic proteins.
JNK: C-Jun amino-terminal kinas
HIF-1α: Hypoxia-inducible factor 1 alpha
HSMECs: Human scar microvascular endothelial cells
GIO: Glucocorticoid-induced osteoporosis
a-C: Amorphous carbo
US: Ultrasound

## CRediT authorship contribution statement

**Yu-ling Liu:** Writing – review & editing, Writing – original draft. **Yue-ming Mei:** Resources. **Jing-qiong Xun:** Conceptualization. **Zhuo-yue Lv:** Formal analysis. **Qian He:** Data curation. **Zhou-bo-ran Liu:** Data curation. **Lin Li:** Resources. **Fen Xie:** Writing – review & editing. **Ru-chun Dai:** Project administration.

## Authorship contribution statement

All authors listed have made a direct and essential contribution to the work, and approved it for publication. YL wrote the original draft and prepared Figs. 1 and Table 1. YM, JX and ZL gave advice on the design of the review ideas and writing. QH, ZL and LL assisted in searching the relevant references and helped with the organization of Table 1. FX and RD reviewed and edited the manuscript; project administration. All authors have read and agreed to the published version of the manuscript.

## Consent for publication

Not applicable.

## Ethics approval and consent to participate

Not applicable.

## Funding

This work was supported by the 10.13039/501100001809National Natural Science Foundation of China (grant numbers 81670804) and 10.13039/501100004735Hunan Provincial Natural Science Foundation (grant numbers 2023JJ30057) and Fundamental Research Funds for the Central Universities of Central South University (grant numbers 2023ZZTS0557) and Clinical Medical Technology Innovation Guiding Project of Hunan Province (2020SK53006).

## Declaration of competing interest

The authors declare that they have no known competing financial interests or personal relationships that could have appeared to influence the work reported in this paper.

## Data Availability

No data was used for the research described in the article.
